# Real‐world experience of using dupilumab and JAK inhibitors to manage pruritus in epidermolysis bullosa pruriginosa

**DOI:** 10.1002/ski2.445

**Published:** 2024-08-19

**Authors:** Ping‐Chen Hou, Wilson Aala, Wei‐Ting Tu, John A. McGrath, Chao‐Kai Hsu

**Affiliations:** ^1^ Department of Dermatology National Cheng Kung University Hospital College of Medicine National Cheng Kung University Tainan Taiwan; ^2^ Institute of Clinical Medicine College of Medicine National Cheng Kung University Tainan Taiwan; ^3^ St John's Institute of Dermatology School of Basic and Medical Biosciences King's College London London UK; ^4^ International Center for Wound Repair and Regeneration National Cheng Kung University Tainan Taiwan

## Abstract

Epidermolysis bullosa pruriginosa (EBP) is a form of dystrophic EB associated with severe pruritus and has skewed Th2 inflammation. Our study suggests that JAK inhibitors may offer superior efficacy compared to dupilumab in treating EBP. Moreover, JAK inhibitors downregulate JAK‐STAT signalling and Th1/2 cell differentiation in lesional skin while not in peripheral blood.

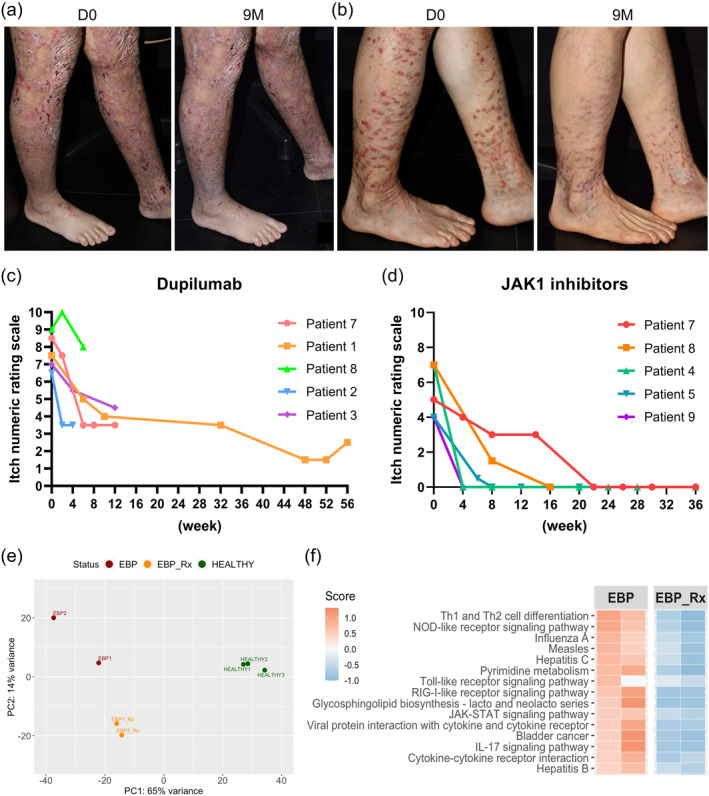

Dear Editor,

Epidermolysis bullosa pruriginosa (EBP) is a form of dystrophic EB characterised by prurigo‐like nodules. It is associated with severe pruritus^1^ and exhibits skewed Th2 inflammation in both blood and lesional skin.^2^ Controlling itch in EBP is a major clinical challenge. Over the last 4 years, anecdotal reports and small case series have highlighted the value of either subcutaneous dupilumab or oral Janus kinase inhibitors (JAKi; baricitinib, upadacitinib and tofacitinib) in reducing EBP itch and improving the patients' skin.^2^, ^3^, ^4^, ^5^, ^6^ Nevertheless, the current literature lacks comparative studies between the two treatments nor have transcriptomic changes following JAKi therapy in EBP been documented to date. Here, we share our experience of using both types of treatment in EBP in clinical practice (Supplementary Table S1) and report gene expression data in EBP skin pre‐ and post‐JAKi.

Following the report by Shehadeh et al. (2020)^3^ of the beneficial impact of dupilumab in EBP, we treated 3 patients with EBP (aged 11, 28 and 43 years) with dupilumab (300 mg subcutaneously every 2 weeks) for up to 14 months (mean: 7.7 months). The average pre‐treatment and post‐treatment itch numeric rating scores (NRS) were 7 (6.5–7.5) and 3.5 (2.5–4.5), respectively. The overall clinical benefit was considered moderate (Figure 1a). Moreover, similar treatment with dupilumab in 2 further subjects with EBP failed to show significant response (see below and Supplementary Table S1). Based on the experience of Jiang et al. (2021)^4^ in using JAKi in EBP, we treated 3 more patients with EBP (aged 18, 52 and 71 years) with abrocitinib (100–200 mg orally daily) for up to 9 months (mean: 5 months). Pruritus in all 3 subjects improved substantially. In the two individuals who recorded itch NRS on abrocitinib, the scores went from 4 and 7 to zero. We then treated 2 further EBP patients (aged 59 and 60 years) who had shown minimal response to dupilumab with JAKi. One patient had responded poorly to 4 doses of dupilumab (itch NRS: 9→8) and was changed to abrocitinib 200 mg/day for 1 month initially, subsequently reduced to 100 mg/day for a further 3 months, with a much better response (itch NRS: 7→0). The other patient initially had a good response following 3 months of dupilumab treatment (itch NRS: 8.5→3.5) but relapsed thereafter. She was re‐treated with dupilumab with minimal response (given 7 months after the first cycle, itch NRS: 6→5). She then changed to upadacitinib 15 mg/day orally for 9 months with a major reduction in pruritus (itch NRS: 5→0). The prurigo lesions flattened, decreased in number and were less erythematous (Figure 1b). One further EBP patient (aged 13 years) had good symptom relief after dupilumab injections for 2 months (no available itch NRS) but was lost to follow‐up. He returned 1 year later with itch recurrence, and abrocitinib 100 mg/day orally was given for 1 month with a rapid response (itch NRS: 4→0) (Supplementary Table S1). Collectively, 6 patients each received dupilumab and JAKi in our study. Of the five dupilumab‐treated patients with available itch NRS, only two experienced a 50% reduction in itch NRS, with none achieving complete relief (NRS = 0). While for JAKi, all 5 patients with available itch scores reached NRS = 0 (Figure 1c, d and Supplementary Table S1). Regarding safety, one patient experienced mild dizziness on abrocitinib and one had a slightly elevated lipid profile on upadacitinib but no major adverse events were reported. No adverse events were reported in the dupilumab arm.

**FIGURE 1:**
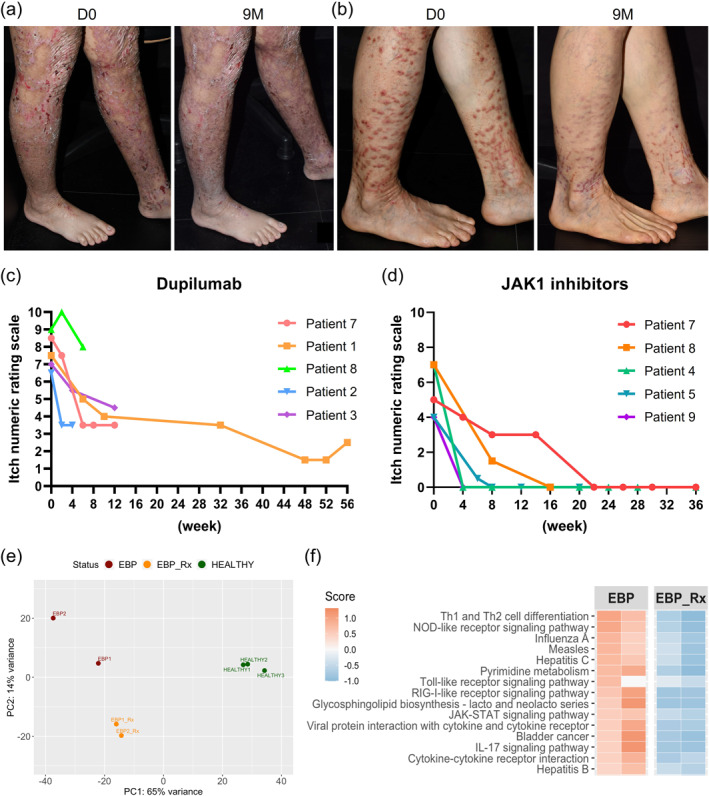
Clinical responses and transcriptomic changes after JAKi in EB pruriginosa. (a) Less erythema and fewer excoriations after 9 months' dupilumab treatment in Patient 1. (b) Reduction and flattening of prurigo‐like lesions after 9 months' upadacitinib treatment in Patient 7. (c) Itch numeric rating score (NRS) in 5 EBP patients treated with dupilumab. (d) Itch NRS in 5 EBP patients treated with JAK1 inhibitors. (e) Principal component analysis reveals that the transcriptomic profiles of JAK inhibitor‐treated skin samples are distinct from pre‐treatment plots (and unrelated, non‐atopic controls). (f) Top 15 enriched pathways in the EBP lesional skin as compared to skin after systemic JAKi treatment.

We also assessed transcriptomic changes following JAKi treatment in the skin and peripheral blood using methods reported elsewhere.^7^ Pre‐ and post‐treatment lesional skin (*n* = 2: 15 weeks after upadacitinib and 8 weeks after abrocitinib) and peripheral blood samples (*n* = 2: 6 and 15 weeks after upadacitinib) were collected. Principal component analysis showed that the transcriptomic profiles of post‐treatment skin specimens were separated from the pre‐treatment samples (Figure 1e). PathfindR enrichment analysis identified Th1/2 cell differentiation as the most enriched pathway in EBP lesional skin compared to post‐treatment skin. Other pathways, such as JAK‐STAT and IL‐17 signalling, were also within the top 15 enriched pathways and were suppressed after JAKi treatment (Figure 1f). However, the peripheral blood transcriptomic analysis failed to identify a significant decrease of Th2 or JAK‐STAT signalling after upadacitinib treatment (data not shown).

Regarding the nature of inflammation in EBP, two other studies immunophenotyped EBP circulating T cells and demonstrated an upregulation of Th2 cells.^2^, ^5^ Recently, two further publications reported transcriptomic data in EBP lesional skin which displayed elevated IL‐4/IL‐13 signalling and Th2 activation pathways similar to the current study.^7^, ^8^ Single‐cell sequencing also highlighted the enrichment of glycolytically active GATA3+ Th2 cells in the affected EBP skin, rationalising the prescription of Th2‐targeted agents.^8^ Of note, our new transcriptomic data highlight that Th1/2 cell differentiation, JAK‐STAT and IL‐17 signalling were suppressed by JAKi in EBP lesional skin but not in the blood. This may imply that the inflammation in EBP is localised to lesional skin instead of a systemic component.

In the literature, both dupilumab and JAKi alleviate itch and prurigo‐like lesions in patients with EBP. Ten studies with a total of 13 cases (ranging from 6 to 52 years old) received dupilumab at a dosage of 300 mg every 2 or 4 weeks for 3–22 months. The therapeutic effects were noted as early as 2–6 weeks in 4 studies and the itch VAS scores reduced from 6 to 10 to 0–5.^3^, ^5^, ^9^ As for JAKi, five studies with 7 EBP patients (ranging from 16 to 40 years old) received tofacitinib (5 mg twice a day), baricitinib (2–4 mg/day) or upadacitinib (15 mg/day) for 4–32 weeks. Reduction in itch and alleviation of skin lesions were typically seen at weeks 2–4, although a single case reported improvement of pruritus as early as day 2 of baricitinib use.^4^ Overall, the itch VAS dropped from 5 to 10 to 1–8.^4^, ^6^, ^10^ However, the treatment efficacy comparison between dupilumab and JAKi in EBP has not been described. In our cohort, JAKi appeared to show better disease control of EBP. The potential superiority of JAKi may be explained by the additional suppression of Th1 and Th17‐related cytokines, which are not targeted directly by dupilumab. Nevertheless, a clear limitation of our study is that we are reporting real‐world experience of these treatments rather than a direct comparative assessment and long‐term follow‐up data are needed. Our study is also limited by a small sample size, lack of randomisation, assessor blinding, patient matching as well as the variable assessment and skin sampling time points. Moreover, the baseline itch NRS scores were slightly higher in the dupilumab‐treated individuals.

In summary, our study suggested that JAKi may offer a superior efficacy compared to dupilumab in treating EBP. Moreover, JAKi downregulates JAK‐STAT signalling and Th1/2 cell differentiation in the lesional skin while the effect is not observed in peripheral blood. Despite the clinical efficacy of JAKi, the possible increased skin cancer risk should be cautioned in the treatment of EB patients.

## CONFLICT OF INTEREST STATEMENT

The authors declare no conflicts of interest.

## AUTHOR CONTRIBUTIONS

**Ping‐Chen Hou**: Data curation (equal); formal analysis (equal); investigation (equal); methodology (equal); project administration (lead); software (equal); validation (equal); visualization (equal); writing – original draft (lead). **Wilson Aala Jr.**: Data curation (equal); formal analysis (equal); investigation (equal); methodology (equal); validation (equal); visualization (equal). **Wei‐Ting Tu**: Data curation (equal); investigation (equal); supervision (equal); writing – review & editing (equal). **John A. McGrath**: Conceptualization (equal); supervision (equal); writing – review & editing (equal). **Chao‐Kai Hsu**: Conceptualization (equal); funding acquisition (equal); investigation (equal); resources (equal); supervision (equal); writing – review & editing (equal).

## FUNDING INFORMATION

National Science and Technology Council, Grant/Award Number: 112‐2314‐B‐006‐020‐MY3; National Cheng Kung University Hospital, Grant/Award Numbers: NCKUH‐11102006, NCKUH‐11209004; Yushan Fellow Program by the Ministry of Education (MOE), Grant/Award Number: MOE‐111‐YSFMN‐0005‐001‐P1.

## ETHICS STATEMENT

This study was approved by the Institutional Review Board of National Cheng Kung University Hospital (IRB number: B‐BR‐110‐090).

## PATIENT CONSENT

Written patient consent for publication was obtained.

## Supporting information

Table S1

## Data Availability

Data are available on request from the authors.
