# Supplementary material for: Real‐world experience of using dupilumab and JAK inhibitors to manage pruritus in epidermolysis bullosa pruriginosa
Source: Skin Health Dis. 2024 Aug 19;4(5):e445. doi: 10.1002/ski2.445 (PMC11442062; doi:10.1002/ski2.445)
Supplement: Supplementary file 1 — Table S1 [file SKI2-4-e445-s001.docx]

**Supplementary Table 1. Demographics and treatment responses of the 9 EBP patients.**

| Treatment | Patient | Sex | Age | *COL7A1* variant | Treatment duration (month) | Pre-treatment  itch NRS score | Post-treatment  itch NRS score |
| --- | --- | --- | --- | --- | --- | --- | --- |
| Dupilumab only | Patient 1 | F | 28 | c.6182G>A (p.Gly2061Glu) | 14 | 7.5 | 2.5 |
|  | Patient 2 | F | 43 | c.4670G>A (p.Gly1557Glu) | 2 | 6.5 | 3.5 |
|  | Patient 3 | M | 11 | c.4670G>A (p.Gly1557Glu) | 7 | 7 | 4.5 |
| JAK inhibitor only (abrocitinib) | Patient 4 | F | 52 | c.4670G>A (p.Gly1557Glu) | 9 | 7 | 0 |
|  | Patient 5 | F | 18 | c.4670G>A (p.Gly1557Glu) | 5 | 4 | 0 |
|  | Patient 6 | F | 71 | c.5318G>T (p.Gly1773Val) | 1 | - | - |
| Dupilumab →  JAK inhibitor | Patient 7 | F | 59 | c.4670G>A (p.Gly1557Glu) | - Poor response to dupilumab (300mg biweekly) (4 doses, itch NRS: 9→8) - Good response to abrocitinib (1-200mg/day) (4 months, itch NRS: 7→0) | | |
|  | Patient 8 | F | 60 | c.7697G>A (p.Gly2566Glu) | - Good response to 1st cycle of dupilumab (3 months, itch NRS: 8.5→3.5) - Poor response to 2nd cycle of dupilumab (1 month, itch NRS: 6→5) - Good response to upadacitinib (15mg/day) (9 months, itch NRS: 5→0) | | |
|  | Patient 9 | M | 13 | c.4670G>A (p.Gly1557Glu) | - With response to dupilumab (300mg biweekly) (2 months, no recorded itch NRS) - Good response to abrocitinib (100mg/day) (1 month, itch NRS: 4→0) | | |

Abbreviations: F – female; JAK – Janus kinase; M – male; NRS – numeric rating scale
